# Healthier Macronutrient Profiles but Higher Risk of Specific Micronutrient Deficiencies: A Cross-Sectional Study of Vegans, Lacto-Ovo-Vegetarians and Omnivores in Northeast China

**DOI:** 10.3390/nu18132109

**Published:** 2026-06-28

**Authors:** Xin Liu, Ang Li, Miaoyu An, Hongyan Wu, Huan Wang, Changbao Sun

**Affiliations:** 1 College of Food and Biological Engineering, Qiqihar University, Qiqihar 161006, China; liuxin200059@163.com (X.L.); liang621liang@126.com (A.L.); anmiaoyv@163.com (M.A.); hljwuhongyan@163.com (H.W.); 2 Branch of Animal Husbandry and Veterinary of Heilongjiang Academy of Agricultural Sciences, Qiqihar 161005, China; whuan0517@126.com

**Keywords:** vegans, omnivores, macronutrient profile, micronutrient deficiency, Northeast China

## Abstract

Background: Data on the nutritional adequacy of unsupplemented vegetarians in Northeast China are limited. Methods: We compared dietary intake, body composition, and serum biomarkers among vegans, lacto-ovo-vegetarians, and omnivores. This cross-sectional study included 356 adults (all diet ≥ 2 years, no supplements). Dietary intake was assessed using a validated semi-quantitative FFQ, body composition by BIA, and serum biomarkers. Kruskal–Wallis tests with Bonferroni correction were used. Results: Vegans had lower BMI (22.0 vs. 24.6 kg/m^2^), body fat (24.5% vs. 28.0%), and visceral fat (0.65 vs. 1.05 L) than omnivores (all *p* < 0.002). Vegans consumed more fiber (38.5 vs. 18.0 g/d) and vitamin C (180 vs. 85 mg/d), but less vitamin B_12_ (0.3 vs. 4.2 μg/d), vitamin D (0.5 vs. 3.2 μg/d), calcium (520 vs. 720 mg/d), iodine (65 vs. 130 μg/d), and selenium (45 vs. 85 μg/d). Serum vitamin B_12_, 25-(OH)D, ferritin, and selenium were significantly lower in vegans, while homocysteine was higher. The proportion of vegans with dietary intake below the recommendation reached 100% for vitamin B_12_ and 97% for vitamin D, whereas omnivores showed excessive sodium (75%) and SFA (70%) intake. Conclusions: In this Northeast China cohort, unsupplemented vegetarian diets offered favorable macronutrient profiles and body composition but were associated with a high prevalence of dietary intakes below recommendations for vitamin B_12_, vitamin D, iodine, selenium, zinc, and calcium. These findings underscore the need for targeted supplementation and food fortification strategies for individuals adhering to plant-based diets without supplement use in this region.

## 1. Introduction

Shifting away from animal-based foods toward plant-predominant eating patterns has gained global momentum, spurred by health considerations, ethical objections to animal farming, and concerns over the environmental toll of high meat production [1,2].

Within China, the proportion of adults identifying as vegetarian has climbed rapidly, especially among younger, city-dwelling, and university-educated groups [3,4,5,6]. In line with this trend, the Chinese government has promoted plant-forward dietary patterns through the updated Dietary Guidelines for Chinese Residents (2022), emphasizing increased consumption of legumes, vegetables, fruits, and whole grains [7].

Numerous large-scale cohort studies have linked well-designed vegetarian and vegan regimens to reduced risks of obesity, type 2 diabetes, hypertension, ischemic heart disease, and certain cancers [8,9,10]. These protective associations are generally credited to lower consumption of saturated fat and cholesterol, coupled with higher intakes of dietary fiber, unsaturated fats, antioxidant vitamins (C and E), folate, magnesium, and various phytochemicals [11,12]. At the same time, concerns persist about whether such diets—particularly when no supplements are used—can provide all essential micronutrients. Animal products are either the sole or primary sources of several nutrients; therefore, unsupplemented plant-based eating may lead to suboptimal status of vitamin B_12_, vitamin D, calcium, iron, zinc, iodine, selenium, and long-chain *n*-3 fatty acids [13,14,15,16].

The northeastern region of China has its own distinctive food culture characterized by high consumption of red meat, pickled vegetables, fermented soy products, and high-temperature cooking with vegetable oils [17,18]. Winters here are long and harsh, with very limited sunlight, which virtually halts endogenous vitamin D synthesis for several months. In addition, the soils in this area are naturally poor in iodine and selenium [19,20]. Against this background, surprisingly little is known about the nutritional status of vegetarians living in Northeast China. Most available studies have been conducted on Buddhist vegetarian communities in Taiwan or Shanghai [21,22,23], who differ substantially from Northeast populations in diet and lifestyle. Moreover, a recent cross-sectional study from Russia reported similar patterns of nutrient inadequacy among vegans and vegetarians, but no such data exist for Northeast China [24]. Therefore, the present study was designed to compare dietary intake, body composition, and serum biomarkers across three groups—vegans, lacto-ovo-vegetarians, and omnivores—in this under-studied region and to offer practical evidence-based suggestions for optimizing plant-based eating.

## 2. Methods

### 2.1. Study Design and Participants

This cross-sectional study was conducted from March 2023 to December 2025 in Northeast China. Participants were recruited via social media postings, university bulletin boards, and community health center announcements. Inclusion criteria: age 18–55 years; adherence to a vegan, lacto-ovo-vegetarian, or omnivorous diet for ≥2 years; self-reported good health without chronic diseases; no use of any dietary supplements for the past 6 months (a deliberate design choice to assess diet-alone adequacy); non-pregnant or lactating; and BMI 18.5–30.0 kg/m^2^. We excluded current smokers, heavy drinkers, persons taking medications that affect nutrient metabolism, those with acute infections, and individuals with known malabsorption syndromes.

Sample size calculation was based on detecting a difference of 140 pmol/L in serum vitamin B_12_ (assuming a standard deviation of 95 pmol/L) with 80% power and a two-sided α = 0.05. To account for three-group comparisons, a Bonferroni-corrected α = 0.017 was applied, yielding a minimum of 66 participants per group. This calculation guided the recruitment target for the primary outcome (serum vitamin B_12_). However, this power calculation was specific to the primary outcome; secondary outcomes (e.g., phase angle, serum zinc, micronutrient intake comparisons) may not be adequately powered for small effect sizes and should, therefore, be interpreted as exploratory.

**Figure 1:**
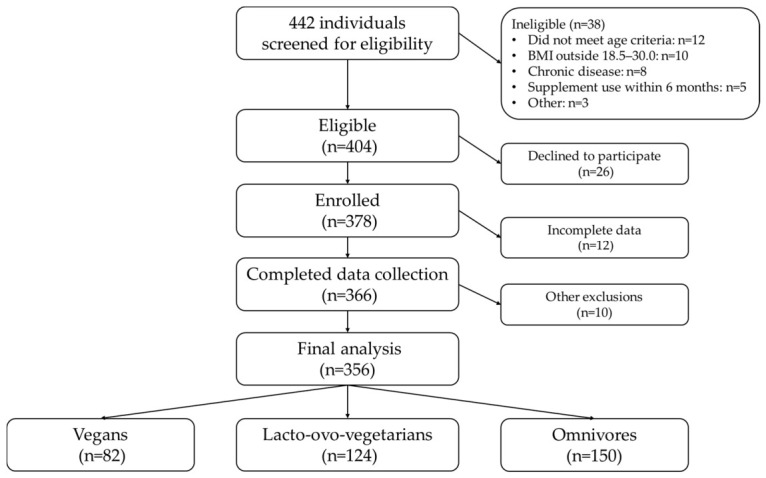
STROBE flow diagram of participant recruitment and exclusion.

Figure 1 presents the STROBE (Strengthening the Reporting of Observational Studies in Epidemiology) flow diagram for participant recruitment and exclusion. After screening 442 individuals, 86 were excluded for the following reasons: 38 did not meet inclusion criteria, 26 declined to participate, 12 had incomplete data, and 10 were excluded for other reasons (e.g., change in dietary pattern during the study period, missing blood samples). The remaining 356 met the criteria and were enrolled: 82 vegans (35 men, 47 women), 124 lacto-ovo-vegetarians (54 men, 70 women), and 150 omnivores (70 men, 80 women). Although group sizes were unequal (82/124/150), all groups exceeded the minimum required sample size of 66 participants per group. The study protocol received approval from the Ethics Committee of Qiqihar University (No. QU-2023-037, date of approval: 20 January 2023) and adhered to the principles of the Declaration of Helsinki. The study is reported in accordance with the STROBE guidelines for cross-sectional studies.

### 2.2. Dietary Assessment

Usual food intake over the year before enrollment was evaluated with a semi-quantitative food frequency questionnaire (SQFFQ) previously validated for vegetarians and omnivores in Northeast China [25]. The SQFFQ included 159 food items aggregated into 22 food groups. For each item, participants reported frequency (from “never” to “≥4 times/day”) and portion size using standard household measures and food photographs. Nutrient calculations used the Chinese Food Composition Tables [26] and were adjusted for total energy intake using the residual method. Validation against three 24 h recalls showed Spearman correlation coefficients of 0.48–0.82, indicating acceptable validity; reproducibility was also satisfactory.

### 2.3. Anthropometric and Body Composition Measurements

Height was measured to the nearest 0.1 cm using a wall-mounted stadiometer (SECA 216, SECA GmbH & Co. KG, Hamburg, Germany) and weight to 0.1 kg on a digital scale (SECA 874, SECA GmbH & Co. KG, Hamburg, Germany). BMI was calculated as weight (kg)/height^2^ (m^2^). Waist circumference was measured at the midpoint between the lowest rib margin and the iliac crest. Body composition was assessed by multi-frequency bioelectrical impedance analysis (SECA mBCA 515, SECA GmbH & Co. KG, Hamburg, Germany), providing fat mass (FM), fat-free mass (FFM), body fat percentage (BF%), visceral adipose tissue (VAT), and phase angle (PhA).

### 2.4. Biochemical Analyses

Fasting venous blood was collected after a 12 h overnight fast. Blood draws occurred across all seasons; the distribution of draw months did not differ significantly among groups (autumn/winter draws: vegans 48%, lacto-ovo-vegetarians 45%, omnivores 50%, *p* = 0.34). Serum lipids, glucose, insulin, high-sensitivity C-reactive protein (hs-CRP), and liver and kidney function markers were measured using an automatic analyzer (AU5800, Beckman Coulter, Inc., Brea, CA, USA). Nutritional biomarkers were assayed as follows: serum vitamin B_12_ by chemiluminescent immunoassay; folate and 25-(OH) D by electrochemiluminescence; ferritin and homocysteine by enzymatic methods; and selenium, zinc, and copper by inductively coupled plasma mass spectrometry (ICP-MS; 8800 Triple Quadrupole, Agilent Technologies, Santa Clara, CA, USA).

### 2.5. Statistical Analysis

Statistical analyses were performed using SPSS 29.0 (IBM Corp., Armonk, NY, USA). Because most continuous variables were non-normally distributed, data were presented as medians with interquartile ranges (IQR). Three-group comparisons employed Kruskal–Wallis H tests. For pairwise comparisons, generalized linear models (GLM) adjusted for age, sex, BMI, physical activity (MET-h/week), and total energy intake (for nutrients) were used, with Bonferroni correction for multiple comparisons (adjusted *p* < 0.017 considered significant). The *P_adj_* values reported in all tables were derived from these GLM models. Dunn’s tests were used for unadjusted exploratory analyses only. Given the large number of outcomes tested (~40 variables), the potential for Type I error was acknowledged. Findings with marginal significance (e.g., phase angle *P_adj_* = 0.041; serum zinc *p* = 0.008) should be interpreted as exploratory. False discovery rate (FDR)-adjusted q-values for all primary comparisons are provided in Supplementary Table S1. The proportion of participants with intake below the recommendation was calculated using Chinese Dietary Reference Intakes (2022) [7]. For nutrients with a Recommended Nutrient Intake (RNI), the proportion with intake <75% of RNI was reported. For nutrients with an Adequate Intake (AI) but no RNI (calcium, iodine), the proportion with intake below the AI was reported. These figures represent the proportion with dietary intake below the recommendation, not the formal prevalence of inadequacy (which would require the EAR cut-point method using multiple days of dietary data). All comparisons across groups were performed using logistic regression adjusted for age, sex, and BMI. Spearman correlations assessed associations between dietary intakes and serum biomarkers; all correlations are exploratory and have not been adjusted for multiple comparisons. All tests were two-sided with *p* < 0.05 indicating statistical significance.

## 3. Results

### 3.1. Participants’ Characteristics

Table 1 summarizes participants’ characteristics. Age (median 34–39 years), sex distribution, education, occupation, and physical activity did not differ significantly among groups (*P_overal_* > 0.05). BMI differed significantly across groups (*P_overall_* < 0.001). Median duration of veganism was 4.5 years for vegans and 5.0 years for lacto-ovo-vegetarians.

### 3.2. Body Composition

Body composition parameters are shown in Table 2. Vegans had significantly lower body weight, BMI, waist circumference, fat mass, body fat percentage, and visceral adipose tissue than omnivores (all *P_adj_* < 0.003). Lacto-ovo-vegetarians also had significantly lower values than omnivores for most parameters. Fat-free mass did not differ significantly among groups. Phase angle was significantly lower in vegans (5.12°) than in omnivores (5.43°, *P_adj_* = 0.041).

### 3.3. Macronutrients and Energy Intake

Table 3 shows energy and macronutrient intakes. Total energy intake did not differ between groups. Vegans had significantly lower protein intake as a percentage of energy (%E) (12.6%E vs. omnivores 15.3%E, *P_adj_* < 0.001) but all groups met the Chinese recommendation (10–15%E). Fat intake (%E) was lowest in vegans (29.7%) and highest in omnivores (36.5%, *P_adj_* = 0.002). Saturated fatty acid intake was dramatically lower in vegans (11.9 g/d) than in omnivores (25.2 g/d, *P_adj_* < 0.001), while polyunsaturated fatty acid intake was higher in vegans (18.7 vs. 12.2 g/d, *P_adj_* < 0.001). The estimated omega-6/omega-3 ratio was significantly higher in vegans (16.3, IQR: 12.4–22.1) than in omnivores (8.3, IQR: 6.3–11.5, *P_adj_* < 0.001). Dietary fiber intake was much higher in vegans (39.6 g/d) than in omnivores (17.0 g/d, *P_adj_* < 0.001), far exceeding the Chinese recommendation.

### 3.4. Micronutrient Intake

Table 4 shows daily micronutrient intakes. Vegans had significantly higher intakes of fiber, vitamin C, vitamin E, folate, magnesium, potassium, and copper than omnivores (all *P_adj_* < 0.001), but significantly lower intakes of vitamin B_12_ (0.3 vs. 4.5 μg/d), vitamin D (0.5 vs. 3.6 μg/d), calcium (513 vs. 737 mg/d), zinc (8.5 vs. 11.5 mg/d), iodine (65 vs. 130 μg/d), and selenium (46 vs. 87 μg/d) (all *P_adj_* < 0.001). Lacto-ovo-vegetarians had intermediate values.

### 3.5. Proportion of Participants with Dietary Intake Below Recommendations

Table 5 shows the proportion of participants with intakes below the Chinese recommendations. Vegans had 100% inadequacy for vitamin B_12_, 97% for vitamin D, 87% for iodine, 70% for selenium, 85% for calcium, and 67% for zinc. Omnivores showed a higher prevalence of excess sodium (75%) and saturated fat (70%). Iron deficiency did not differ among groups (*p* = 0.325), but serum ferritin was significantly lower in vegans (see Section 3.6).

### 3.6. Serum Biomarkers

Serum biomarkers are presented in Table 6. Vegans had significantly lower serum vitamin B_12_ (186 pmol/L) than omnivores (336 pmol/L, *P_adj_* < 0.001) and higher homocysteine (14.3 vs. 10.1 μmol/L, *P_adj_* < 0.001). Serum 25-(OH) D was lowest in vegans (36 nmol/L) and highest in omnivores (63 nmol/L, *P_adj_* < 0.001). Serum ferritin was significantly lower in vegans (33 μg/L) than in omnivores (86 μg/L, *P_adj_* < 0.001), despite vegans having the highest dietary iron intake. Serum selenium and zinc were also significantly lower in vegans. Lipid profiles were most favorable in vegans: total cholesterol (3.7 vs. 4.8 mmol/L), LDL-C (2.2 vs. 3.0 mmol/L), and triglycerides (0.75 vs. 1.20 mmol/L) were lower, while HDL-C was higher (1.58 vs. 1.36 mmol/L) (all *P_adj_* < 0.01). hs-CRP did not differ significantly.

### 3.7. Correlations Between Dietary Intake and Serum Biomarkers

Spearman correlations (Table 7) showed strong positive correlations between dietary and serum vitamin B_12_ (*r* = 0.65) and selenium (*r* = 0.53). Dietary vitamin B_12_ was inversely correlated with homocysteine (*r* = −0.47). Dietary vitamin D was moderately correlated with 25(OH)D (*r* = 0.48). Dietary iron was not correlated with ferritin (*r* = 0.07, *p* = 0.142), illustrating the bioavailability issue. Fiber intake was weakly inversely correlated with hs-CRP (*r* = −0.25, *p* = 0.003).

## 4. Discussion

This cross-sectional study provided a comprehensive assessment of the nutritional status of unsupplemented vegans, lacto-ovo-vegetarians, and omnivores in Northeast China.

### 4.1. Body Composition and Cardiovascular Benefits

The vegans and, to a lesser extent, lacto-ovo-vegetarians had substantially more favorable body composition and cardiovascular risk profiles than omnivores, with lower BMI, body fat, visceral fat, total and LDL cholesterol, triglycerides, and higher HDL cholesterol. These results align with large Western cohorts [8,9] and extend them to the distinct dietary and environmental context of Northeast China. These differences remained significant after adjustment for age, sex, and physical activity, supporting a genuine dietary effect. The magnitude of difference in BMI (2.7 kg/m^2^) and LDL-C (0.8 mmol/L) was clinically meaningful; in previous cohort studies and randomized trials, an LDL-C reduction of this magnitude has been associated with an estimated 15–20% reduction in cardiovascular risk [27,28], suggesting that the observed differences between vegans and omnivores in our study were clinically meaningful. The phase angle data (5.12° in vegans vs. 5.43° in omnivores, *p* = 0.041) suggested subtle differences in cellular integrity. However, both values fall within the normal range for healthy adults (5–7°), and the absolute difference is small (0.31°). This difference may partly reflect intergroup differences in body composition—particularly lower fat mass in vegans—rather than a direct indication of clinically meaningful cellular damage. Moreover, this comparison was secondary and exploratory; after FDR correction for multiple comparisons, the q-value for this finding was 0.115, which does not reach the conventional FDR significance threshold (q < 0.05); therefore, this finding should be interpreted with caution and requires confirmation in future studies with larger sample sizes.

### 4.2. Macronutrient and Fiber Adequacy

Despite common public concerns about protein inadequacy in plant-based diets, vegan protein intake (12.6%E) met the Chinese RNI (10–15%E) [7]; however, the quality and amino acid profile of plant proteins deserve attention [29,30]. The main protein sources in the vegan diet included soy products (tofu, soy milk, tempeh), seitan, legumes (lentils, chickpeas), nuts, and whole grains. Soy protein is of high quality with a digestible indispensable amino acid score (DIAAS) comparable to animal proteins, while other plant proteins may be limited in lysine or methionine; complementary protein strategies can address these limitations (Supplementary Table S2). Fiber intake in vegans (39.6 g/d) far exceeded national recommendations (25–35 g/d) [7], which might protect against colorectal cancer, cardiovascular disease, and type 2 diabetes [31,32]. The low SFA and high PUFA intake in vegans was commendable. However, the estimated omega-6/omega-3 ratio was significantly higher in vegans (16.3) than in omnivores (8.3). A high omega-6/omega-3 ratio has been associated with increased pro-inflammatory states and may be a marker of elevated cardiovascular risk. This ratio warrants attention in dietary guidance for vegans, who may benefit from increased intake of alpha-linolenic acid-rich sources such as flaxseed, chia seeds, and walnuts.

### 4.3. Critical Micronutrient Deficiencies

While the cardiovascular advantages of plant-based diets are well recognized, our data reveal strikingly high rates of micronutrient inadequacy, especially among vegans. Importantly, these deficits are not simply a consequence of avoiding animal products; they are exacerbated by local environmental and behavioral factors specific to Northeast China. Furthermore, because the supplement users were excluded, the high figures (e.g., 100% for B_12_, 97% for vitamin D) reflect the diet-alone scenario and should not be extrapolated to all vegans, many of whom use supplements in practice. Vitamin B_12_ inadequacy was virtually universal among vegans (100%) and very high among lacto-ovo-vegetarians (79%), accompanied by low serum vitamin B_12_ and elevated homocysteine. In China, plant-based milks are rarely fortified with vitamin B_12_, and mandatory fortification of staples was absent [33,34]; therefore, supplementation was the only practical solution. Vitamin D inadequacy was also extremely common, especially in vegans (97%), due to the high latitude where cutaneous synthesis was impossible in winter and the lack of mandatory vitamin D fortification in China [35,36]. Unlike many Western countries, China has no mandatory vitamin D fortification of dairy or plant-based milks. Consequently, all residents—regardless of diet—are at risk, but vegans are most severely affected. Iodine and selenium deficiencies were particularly prominent in this study. Iodine and selenium are naturally low in the soils of Northeast China. In our study, 87% of vegans had inadequate iodine intake and 70% had inadequate selenium intake. Vegans often reduce iodized salt use (replacing it with “natural” sea salt) and do not consume selenium-rich animal products (fish, organ meats). Iodine and selenium deficiencies can impair thyroid function, especially when both are low [37,38]. However, the urinary iodine was not measured; these iodine findings are based on SQFFQ estimates and should be interpreted with particular caution. A daily supplementation of 150 μg of iodine and 50–100 μg of selenium is, therefore, reasonable for vegans living in this region. Calcium inadequacy (85%) and zinc inadequacy (67%) were also major concerns for vegans in this region. Furthermore, the bioavailability of plant-derived calcium and zinc was inhibited by phytate and oxalate, necessitating the selection of low-oxalate calcium sources (such as calcium-set tofu, bok choy) and the use of soaking, sprouting, or fermentation to reduce phytate content. Consistent with the lower dietary zinc intake, serum zinc was also significantly lower in vegans compared to omnivores (*P_adj_* = 0.008). However, after FDR correction for multiple comparisons, the q-value was 0.062, which was above the conventional FDR significance threshold (q < 0.05); thus, this finding should be considered exploratory and interpreted with caution. Vegans had the highest dietary iron intake (18.6 mg/d) and yet the lowest serum ferritin (33 µg/L); a classic demonstration of poor non-heme iron bioavailability due to phytate and polyphenols, despite ample vitamin C [39,40]. In this study, the absence of a correlation between dietary iron and ferritin (*r* = 0.07, *p* = 0.142) confirmed that iron status in vegetarians could not be assessed from intake alone; ferritin measurement was essential. It was recommended to consume iron-rich foods together with vitamin C and to use traditional fermentation (e.g., in tofu, tempeh) to improve iron absorption. Nearly two-thirds of omnivores had sodium intake exceeding the UL (2300 mg/d), and 70% exceeded the SFA recommendation (<10%E). This reflected the typical Northeast Chinese diet: rich in processed meat products, pickled vegetables, fried foods, and cooking oils [4,41,42]. These factors contribute to hypertension and cardiovascular disease. Our findings were consistent with national data showing high sodium and fat intakes in northern Chinese populations.

### 4.4. Comparison with Other Studies

Compared with Western vegetarian studies [43], our participants had lower vitamin B_12_ and iodine intakes, likely due to less fortification and lower use of iodized salt, but higher fiber and vitamin C intakes, reflecting the traditional northeast diet rich in vegetables and fermented soy products [18]. Compared with Taiwanese Buddhist vegetarians [21], our participants had poorer calcium and vitamin D status, consistent with lower dairy consumption and less sun exposure. Our results closely parallel those of Galchenko et al. (2025) [24], who examined vegans, lacto-ovo-vegetarians, and omnivores in Moscow. Both studies found that vegans had superior lipid profiles and lower body weight but severe deficiencies in vitamin B_12_, vitamin D, and selenium. However, important differences exist: our participants had lower vitamin D status (median 36 vs. ~50 nmol/L), likely due to Northeast China’s higher latitude (though Moscow is further north, its winter sun exposure is similarly limited; the difference may arise from lifestyle factors). Moreover, our vegan group had higher fiber intake (39.6 vs. ~36 g/d) but lower calcium intake (513 vs. ~776 mg/d), reflecting lower dairy consumption and fewer calcium-fortified foods in China. These comparisons highlight the importance of regional dietary and policy contexts in shaping nutritional outcomes.

## 5. Strengths and Limitations

The main strengths of this study include the following: (1) This was a comprehensive evaluation of unsupplemented vegans, lacto-ovo-vegetarians, and omnivores in Northeast China, combining validated SQFFQ, BIA, and multiple biomarkers. (2) Exclusion of supplement users allowed for the evaluation of dietary adequacy without confounding variables. (3) We rigorously adjusted for multiple confounders and multiple comparisons.

Limitations: (1) The cross-sectional design precluded causal inference. (2) Despite validation of the SQFFQ, recall bias might still exist. (3) Urinary iodine, the gold standard indicator of iodine status, was not measured. (4) Convenience/volunteer sampling likely recruited a more health-conscious population, which may limit generalizability.

## 6. Conclusions

In this Northeast China cohort, unsupplemented vegetarian and vegan diets were associated with favorable macronutrient profiles, body composition, and cardiovascular biomarkers. However, high proportions of participants—especially vegans—had dietary intakes below recommendations for vitamin B_12_ (100% of vegans), vitamin D (97%), iodine (87%), selenium (70%), zinc (67%), and calcium (85%). These figures reflect the diet-alone scenario among unsupplemented individuals and should not be extrapolated to all vegans, many of whom use supplements in practice. These coexisting benefits and risks indicated that plant-based diets could not be simplistically labeled as “completely healthy.” Responsible practice required targeted supplementation (vitamin B_12_ 25–100 μg/d, vitamin D 15–25 μg/d in winter, iodine 150 μg/d, selenium 50–100 μg/d) and informed food choices. Public health policies should update dietary guidelines and promote food fortification to support the growing number of plant-based dieters and prevent subclinical micronutrient deficiencies.

## Figures and Tables

**Table 1:** Baseline characteristics of participants (median, IQR or n (%)).

Characteristics	Vegans (*n* = 82)	Lacto-Ovo-Vegetarians (*n* = 124)	Omnivores (*n* = 150)	*P_overall_*
Age (years)	34 (24–50)	36 (23–52)	39 (18–55)	0.142
Sex: male *n* (%)	35 (42.7)	54 (43.5)	70 (46.7)	0.089
BMI (kg/m^2^)	22.0 (19.3–23.9)	23.1 (21.5–24.7)	24.7 (22.8–26.6)	<0.001
Education: ≥college n (%)	65 (79.3)	91 (73.4)	98 (65.3)	0.128
Physical activity (MET-h/wk)	28.6 (16.0–41.0)	26.1 (14.0–41.0)	24.1 (12.0–37.0)	0.112
Diet duration (years)	4.5 (2.5–8.0)	5.0 (3.0–8.0)	—	—

*P_overall_* from Kruskal–Wallis H test.

**Table 2:** Body composition parameters (median, IQR).

Parameter	Vegans (*n* = 82)	Lacto-Ovo-Vegetarians (*n* = 124)	Omnivores (*n* = 150)	*P_overall_*	*P_adj_* (V vs. O)	*P_adj_* (Veg vs. O)
Body weight (kg)	60.3 (53.5–68.0)	62.8 (57.2–73.4)	68.8 (60.3–78.6)	<0.001	<0.001	0.008
BMI (kg/m^2^)	22.0 (20.2–23.9)	23.2 (21.6–24.8)	24.7 (22.9–27.8)	<0.001	<0.001	0.006
Waist circumference (cm)	74.6 (67.0–79.5)	76.5 (70.5–82.4)	80.5 (74.5–88.0)	<0.001	<0.001	0.012
Fat mass (kg)	14.8 (10.9–19.0)	17.2 (12.8–21.6)	19.7 (15.4–25.1)	<0.001	<0.001	0.015
Body fat (%)	24.6 (20.0–29.0)	26.6 (22.0–31.0)	28.1 (24.0–33.0)	0.002	0.003	0.072
Fat-free mass (kg)	45.1 (40.2–49.6)	46.0 (41.3–50.9)	47.3 (42.4–52.3)	0.089	0.312	0.642
Visceral adipose tissue (L)	0.64 (0.45–0.95)	0.80 (0.54–1.12)	1.07 (0.71–1.47)	<0.001	<0.001	0.008
Phase angle (°)	5.12 (4.70–5.55)	5.26 (4.91–5.63)	5.43 (5.05–5.85)	0.041	0.041	0.186
Basal metabolic rate (kcal/d)	1354 (1270–1460)	1395 (1310–1490)	1411 (1330–1540)	0.065	—	—

*P_overall_* from Kruskal–Wallis H test. *P_adj_* from generalized linear models adjusted for age, sex, BMI, and physical activity (MET-h/week), with Bonferroni correction. FDR-adjusted q-values for all comparisons are provided in Supplementary Table S1. Abbreviations: V, vegans; Veg, lacto-ovo-vegetarians; O, omnivores.

**Table 3:** Daily energy and macronutrient intakes (median, IQR).

Nutrient	Vegans (*n* = 82)	Lacto-Ovo-Vegetarians (*n* = 124)	Omnivores (*n* = 150)	*P_overall_*	*P_adj_* (V vs. O)	*P_adj_* (Veg vs. O)
Energy (kcal/d)	1985 (1675–2365)	2060 (1755–2490)	2165 (1835–2650)	0.087	—	—
Protein (g/d)	64 (55–78)	69 (58–86)	87 (70–99)	<0.001	<0.001	0.002
Protein (%E)	12.6 (10.4–14.7)	13.5 (11.6–15.0)	15.3 (13.6–17.3)	<0.001	<0.001	0.008
Fat (g/d)	63 (50–82)	75 (61–89)	88 (68–112)	<0.001	<0.001	0.006
Fat (%E)	29.7 (26.2–33.8)	32.6 (27.4–36.8)	36.5 (32.9–39.2)	0.002	0.002	0.058
SFA (g/d)	11.9 (9.3–16.5)	18.0 (15.1–24.2)	25.2 (21.1–32.2)	<0.001	<0.001	<0.001
PUFA (g/d)	18.7 (14.1–24.3)	14.6 (12.1–19.6)	12.2 (9.1–15.8)	<0.001	<0.001	0.062
MUFA (g/d)	19.8 (14.8–26.1)	23.5 (19.2–29.6)	28.2 (22.7–35.8)	<0.001	<0.001	0.004
Omega-6/Omega-3 ratio	16.3 (12.4–22.1)	12.8 (9.5–17.6)	8.3 (6.3–11.5)	<0.001	<0.001	0.002
Carbohydrates (g/d)	275 (236–328)	262 (225–316)	245 (205–295)	0.008	0.004	0.078
Carbohydrates (%E)	56.6 (52.1–61.3)	51.2 (46.8–55.8)	45.7 (42.2–49.7)	<0.001	<0.001	0.002
Dietary fiber (g/d)	39.6 (31.0–49.0)	27.0 (21.0–34.6)	17.0 (13.2–22.0)	<0.001	<0.001	<0.001
Cholesterol (mg/d)	13 (4–34)	96 (44–165)	325 (245–430)	<0.001	<0.001	<0.001

*P_overall_* from Kruskal–Wallis H test (energy-adjusted for all nutrients except energy). *P_adj_* from generalized linear models adjusted for age, sex, BMI, physical activity, and energy intake (for nutrients), with Bonferroni correction. FDR-adjusted q-values for all comparisons are provided in Supplementary Table S1. Abbreviations: %E, percentage of total energy; SFA, saturated fatty acids; PUFA, polyunsaturated fatty acids; MUFA, monounsaturated fatty acids; V, vegans; Veg, lacto-ovo-vegetarians; O, omnivores.

**Table 4:** Daily micronutrient intakes (median, IQR).

Micronutrients	Vegans (*n* = 82)	Lacto-Ovo-Vegetarians (*n* = 124)	Omnivores (*n* = 150)	*P_overall_*	*P_adj_* (V vs. O)
Vitamin B_12_ (μg/d)	0.3 (0.1–0.6)	1.2 (0.6–1.9)	4.5 (2.8–6.2)	<0.001	<0.001
Vitamin D (μg/d)	0.5 (0.2–1.1)	1.4 (0.8–2.7)	3.6 (1.7–5.5)	<0.001	<0.001
Folate (μg DFE/d)	522 (410–655)	445 (350–565)	370 (310–490)	<0.001	<0.001
Vitamin C (mg/d)	185 (120–256)	126 (92–194)	84 (62–136)	<0.001	<0.001
Vitamin E (mg α-TE/d)	18.6 (14.2–24.7)	13.4 (11.2–18.8)	10.5 (8.8–15.9)	<0.001	<0.001
Calcium (mg/d)	513 (410–657)	682 (556–830)	737 (576–891)	<0.001	<0.001
Magnesium (mg/d)	486 (388–592)	378 (310–465)	310 (255–386)	<0.001	<0.001
Iron (mg/d)	18.6 (15.3–22.9)	16.1 (13.4–19.5)	15.1 (12.0–18.5)	0.006	0.003
Zinc (mg/d)	8.4 (6.7–10.5)	9.7 (7.5–12.8)	11.5 (9.3–14.8)	<0.001	<0.001
Iodine (μg/d)	65 (45–95)	95 (73–125)	130 (105–180)	<0.001	<0.001
Selenium (μg/d)	46 (33–64)	65 (54–85)	87 (68–115)	<0.001	<0.001
Potassium (mg/d)	3250 (2700–3850)	2750 (2350–3400)	2250 (2000–2850)	<0.001	<0.001
Sodium (mg/d)	2650 (2150–3400)	2850 (2300–3550)	3100 (2550–3800)	0.018	0.068
Copper (mg/d)	1.9 (1.3–2.4)	1.4 (1.1–1.9)	1.2 (0.9–1.7)	<0.001	<0.001

*P_overall_* from Kruskal–Wallis H test. *P_adj_* from generalized linear models adjusted for age, sex, BMI, physical activity, and energy intake, with Bonferroni correction. FDR-adjusted q-values for all comparisons are provided in Supplementary Table S1. Abbreviations: V, vegans; O, omnivores. Only vegans vs. omnivores showed brevity; all comparisons with vegetarians were also significant except sodium.

**Table 5:** Proportion of participants with dietary intake below recommendations by dietary group (%).

Nutrient (Criteria)	Vegans	Lacto-Ovo-Vegetarians	Omnivores	*P_adj_*
Vitamin B_12_ (<75% RNI)	100	79	23	<0.001
Vitamin D (<75% RNI)	97	84	64	<0.001
Folate (<75% RNI)	11	29	47	0.007
Vitamin C (<75% RNI)	4	16	28	0.002
Calcium (<AI)	85	56	46	<0.001
Iron (<75% RNI)	19	23	25	0.325
Zinc (<75% RNI)	67	41	18	<0.001
Iodine (<AI)	87	65	40	<0.001
Selenium (<75% RNI)	70	37	16	<0.001
Excess Sodium (>UL)	27	47	75	<0.001
Excess SFA (>10%E)	8	36	70	<0.001

Adjusted for age, sex, and BMI; logistic regression. Abbreviations: RNI, recommended nutrient intake; AI, adequate intake; UL, tolerable upper intake level; SFA, saturated fatty acids; E, energy.

**Table 6:** Serum biomarkers (median, IQR).

Biomarker	Vegans (*n* = 82)	Lacto-Ovo-Vegetarians (*n* = 124)	Omnivores (*n* = 150)	*P_overall_*	*P_adj_* (V vs. O)
Vitamin B_12_ (pmol/L)	186 (136–235)	247 (195–318)	336 (263–419)	<0.001	<0.001
Folate (nmol/L)	28.6 (21.7–36.5)	24.2 (17.5–32.7)	20.0 (16.0–28.9)	<0.001	<0.001
25-(OH) D (nmol/L)	36 (28–56)	47 (34–62)	63 (44–78)	<0.001	<0.001
Homocysteine (μmol/L)	14.3 (11.6–18.5)	12.0 (9.4–15.3)	10.1 (8.5–12.8)	<0.001	<0.001
Ferritin (μg/L)	33 (21–67)	55 (30–93)	86 (52–137)	<0.001	<0.001
Selenium (μg/L)	68 (55–85)	80 (67–94)	93 (78–112)	<0.001	<0.001
Zinc (μmol/L)	11.6 (10.3–14.2)	12.3 (11.5–16.7)	13.2 (10.5–16.8)	0.008	0.008
Total cholesterol (mmol/L)	3.7 (3.4–4.6)	4.3 (3.8–5.1)	4.8 (4.1–5.6)	<0.001	<0.001
HDL-C (mmol/L)	1.58 (1.25–1.78)	1.50 (1.31–1.72)	1.36 (1.18–1.65)	0.002	0.002
LDL-C (mmol/L)	2.2 (1.5–2.7)	2.6 (2.0–3.2)	3.0 (2.4–3.7)	<0.001	<0.001
Triglycerides (mmol/L)	0.75 (0.65–1.15)	1.05 (0.80–1.45)	1.20 (0.95–1.65)	0.006	0.006
hs-CRP (mg/L)	0.95 (0.65–1.90)	1.10 (0.68–2.18)	1.35 (0.72–2.46)	0.092	-

*P_overall_* from Kruskal–Wallis H test. *P_adj_* from generalized linear models adjusted for age, sex, BMI, and physical activity, with Bonferroni correction. FDR-adjusted q-values for all comparisons are provided in Supplementary Table S1. Abbreviations: V, vegans; O, omnivores.

**Table 7:** Spearman correlation coefficients between dietary intake and serum biomarkers.

Dietary Component	Serum Biomarker	*r*	*p*
Vitamin B_12_ intake	Serum vitamin B_12_	0.65	<0.001
Vitamin B_12_ intake	Homocysteine	−0.47	<0.001
Vitamin D intake	25(OH)D	0.48	<0.001
Folate intake	Serum folate	0.39	<0.001
Iron intake	Serum ferritin	0.07	0.142
Selenium intake	Serum selenium	0.53	<0.001
Zinc intake	Serum zinc	0.34	<0.001
Fiber intake	hs-CRP	−0.25	0.003

Spearman correlations were exploratory and have not been adjusted for multiple comparisons. FDR-adjusted q-values are provided in Supplementary Table S1.

## Data Availability

The data presented in this study are available on request from the corresponding author.

## Supplementary Materials

The following supporting information can be downloaded at: https://www.mdpi.com/article/10.3390/nu18132109/s1, Table S1: FDR-adjusted q-values for primary comparisons; Table S2: Major food sources of key nutrients based on the SQFFQ.
