# Supplementary material for: Healthier Macronutrient Profiles but Higher Risk of Specific Micronutrient Deficiencies: A Cross-Sectional Study of Vegans, Lacto-Ovo-Vegetarians and Omnivores in Northeast China
Source: Nutrients. 2026 Jun 28;18(13):2109. doi: 10.3390/nu18132109 (PMC13363170; doi:10.3390/nu18132109)
Supplement: Supplementary file 1 [file nutrients-18-02109-s001.zip › nutrients-4366320-supplementary.pdf]

Supplementary Table S1. FDR-adjusted q-values for primary comparisons.

Table S1-A. Body composition parameters.

Parameter	Comparison	P_{adj} (Bonferroni)	FDR q-value
Body weight	Vegans vs. Omnivores	<0.001	<0.001
Body weight	Lacto-ovo-vegetarians vs. Omnivores	0.008	0.008
BMI	Vegans vs. Omnivores	<0.001	<0.001
BMI	Lacto-ovo-vegetarians vs. Omnivores	0.006	0.006
Waist circumference	Vegans vs. Omnivores	<0.001	<0.001
Waist circumference	Lacto-ovo-vegetarians vs. Omnivores	0.012	0.012
Fat mass	Vegans vs. Omnivores	<0.001	<0.001
Fat mass	Lacto-ovo-vegetarians vs. Omnivores	0.015	0.015
Body fat percentage	Vegans vs. Omnivores	0.003	0.003
Body fat percentage	Lacto-ovo-vegetarians vs. Omnivores	0.072	0.072*
Fat-free mass	Vegans vs. Omnivores	0.312	0.312
Visceral adipose tissue	Vegans vs. Omnivores	<0.001	<0.001
Visceral adipose tissue	Lacto-ovo-vegetarians vs. Omnivores	0.008	0.008
Phase angle	Vegans vs. Omnivores	0.041	0.115*

Table S1-B. Macronutrient intakes (energy-adjusted).

Nutrient	Comparison	P_{adj} (Bonferroni)	FDR q-value
Protein (g/d)	Vegans vs. Omnivores	<0.001	<0.001
Protein (%E)	Vegans vs. Omnivores	<0.001	<0.001
Fat (g/d)	Vegans vs. Omnivores	<0.001	<0.001
Fat (%E)	Vegans vs. Omnivores	0.002	0.002
SFA (g/d)	Vegans vs. Omnivores	<0.001	<0.001
PUFA (g/d)	Vegans vs. Omnivores	<0.001	<0.001
MUFA (g/d)	Vegans vs. Omnivores	<0.001	<0.001
Omega-6/Omega-3 ratio	Vegans vs. Omnivores	<0.001	<0.001
Carbohydrates (g/d)	Vegans vs. Omnivores	0.004	0.004
Carbohydrates (%E)	Vegans vs. Omnivores	<0.001	<0.001
Dietary fiber (g/d)	Vegans vs. Omnivores	<0.001	<0.001
Cholesterol (mg/d)	Vegans vs. Omnivores	<0.001	<0.001

Table S1-C. Micronutrient intakes (energy-adjusted) – Vegans vs. Omnivores.

Micronutrient	P_{adj} (Bonferroni)	FDR q-value
Vitamin B_{12}	<0.001	<0.001
Vitamin D	<0.001	<0.001
Folate	<0.001	<0.001

Vitamin C	<0.001	<0.001
Vitamin E	<0.001	<0.001
Calcium	<0.001	<0.001
Magnesium	<0.001	<0.001
Iron	0.003	0.003
Zinc	<0.001	<0.001
Iodine	<0.001	<0.001
Selenium	<0.001	<0.001
Potassium	<0.001	<0.001
Sodium	0.068	0.134*
Copper	<0.001	<0.001

Table S1-D. Serum biomarkers – Vegans vs. Omnivores.

**Biomarker**	**P_{adj} (Bonferroni)**	**FDR q-value**
Vitamin B_{12}	<0.001	<0.001
Folate	<0.001	<0.001
25(OH)D	<0.001	<0.001
Homocysteine	<0.001	<0.001
Ferritin	<0.001	<0.001
Selenium	<0.001	<0.001
Zinc	0.008	0.062*
Total cholesterol	<0.001	<0.001
HDL-C	0.002	0.002
LDL-C	<0.001	<0.001
Triglycerides	0.006	0.006
hs-CRP	0.092	0.165*

Table S1-E. Spearman correlations (exploratory).

**Dietary component**	**Serum biomarker**	***P* value**	**FDR q-value**
Vitamin B_{12} intake	Serum vitamin B_{12}	<0.001	<0.001
Vitamin B_{12} intake	Homocysteine	<0.001	<0.001
Vitamin D intake	25(OH)D	<0.001	<0.001
Folate intake	Serum folate	<0.001	<0.001
Iron intake	Serum ferritin	0.142	0.213*
Selenium intake	Serum selenium	<0.001	<0.001
Zinc intake	Serum zinc	<0.001	<0.001
Fiber intake	hs-CRP	0.003	0.003

Notes: $q < 0.05$ indicates statistical significance at the FDR level. Entries marked with * ($q \geq 0.10$) were considered exploratory and should be interpreted with caution.

Supplementary Table S2. Major food sources of key nutrients based on the SQFFQ.

Nutrient	Major plant-based sources	Major animal-based sources
Vitamin B_{12}	Fortified plant milks, nutritional yeast	Meat, fish, eggs, dairy
Vitamin D	Some mushrooms	Fatty fish, egg yolks, fortified dairy
Calcium	Calcium-set tofu, bok choy, kale, broccoli, almonds, tahini	Dairy products, fish with bones
Iodine	Iodized salt, seaweed	Dairy, fish, eggs
Selenium	Brazil nuts, sunflower seeds, whole grains	Fish, meat, eggs
Zinc	Legumes, nuts, seeds, whole grains	Meat, shellfish, dairy
Iron (non-heme)	Legumes, dark leafy greens, whole grains, fortified cereals	Meat, poultry, fish (heme iron)
Vitamin C	Citrus fruits, bell peppers, broccoli, kiwi	Minimal
Dietary fiber	Whole grains, legumes, vegetables, fruits, nuts, seeds	None
Omega-3 (ALA)	Flaxseed, chia seeds, walnuts, hemp seeds	Fish (EPA/DHA)
